# Mid-Arm Circumference and All-Cause, Cardiovascular, and Cancer Mortality among Obese and Non-Obese US Adults: the National Health and Nutrition Examination Survey III

**DOI:** 10.1038/s41598-017-02663-7

**Published:** 2017-05-23

**Authors:** Li-Wei Wu, Yuan-Yung Lin, Tung-Wei Kao, Chien-Ming Lin, Chung-Ching Wang, Gia-Chi Wang, Tao-Chun Peng, Wei-Liang Chen

**Affiliations:** 1 Division of Family Medicine, Department of Family and Community Medicine, Tri-Service General Hospital, and School of Medicine, National Defense Medical Center, Taipei, Taiwan Republic of China; 2 Division of Geriatric Medicine, Department of Family and Community Medicine, Tri-Service General Hospital, and School of Medicine, National Defense Medical Center, Taipei, Taiwan Republic of China; 3 Graduate Institute of Medical Sciences, National Defense Medical Center, Taipei, Taiwan Republic of China; 4 Department of Otolaryngology–Head and Neck Surgery, Tri-Service General Hospital; and School of Medicine, National Defense Medical Center, Taipei, Taiwan Republic of China; 5 Department of Pediatrics, Tri-Service General Hospital; and School of Medicine, National Defense Medical Center, Taipei, Taiwan Republic of China

## Abstract

Epidemiological studies have shown that mid-arm circumference (MAC) can be used to predict death risk and malnutrition. We performed a retrospective observational study involving 11,958 US participants aged 20–90 years from the National Health and Nutrition Examination Survey III, 1988–1994, to determine the correlation between MAC and all-cause, cardiovascular, and cancer mortality risk in the obese and non-obese population. Death certificate data were obtained up to 2006. The participants were divided into three groups on the basis of body mass index: 19 ≤ BMI < 25 kg/m^2^ (normal weight group), 25 ≤ BMI < 30 kg/m^2^ (overweight group) and BMI ≥ 30 kg/m^2^ (obesity group); each group was then divided into three subgroups depending on their MAC level. In the non-obese population, MAC was inversely associated with all-cause mortality; specifically, in the normal weight group, the multivariate-adjusted hazard ratio of the T3 (29.6–42.0) cm subgroup was 0.72 (95% confidence interval: 0.58–0.90) when compared with the T1 (18.0–27.2) cm, while the multivariate-adjusted hazard ratio of the T2 (27.3–29.5) cm subgroup was 0.76 (95% confidence interval: 0.64–0.91) when compared with the T1 (18.0–27.2) cm subgroup. The results indicate that MAC is inversely associated with all-cause mortality in non-obese individuals in the United States.

## Introduction

Numerous epidemiological studies have shown that obesity is associated with a high risk of hypertension, hypercholesterolemia, type 2 diabetes mellitus, cardiovascular disease, and overall death^1^. Indeed, in the National Health and Nutrition Examination Survey (NHANES) 1999–2004, obesity and overweightness were highly prevalent; in fact, they have become an epidemic among older adults in the United States (US)^2^. Body mass index (BMI: a person’s weight in kilograms divided by the square of their height in meters) is widely used to screen for overweightness and risk of obesity-related disease^3^. Troiano *et al*. reported a U-shaped relationship between mortality and BMI, indicating that both high and low BMI are linked to a higher risk of death^4^. However, BMI has several limitations as a predictor of mortality: for instance, it does not distinguish between lean and fat mass, nor does it take into account relative fat distribution^5–7^.

Recently, several anthropometric parameters have been used to complement the BMI-based estimation of obesity-related risk and nutritional status; namely, mid-arm circumference (MAC), waist circumference, hip circumference, and skinfold thickness. In this regard, several studies have shown that MAC can be used to reliably estimate the risk of death, particularly in infants and children^8, 9^. Moreover, Mason *et al*.^10^ and Landi *et al*.^11^ reported that MAC was inversely associated with all-cause mortality in adults^10, 11^. However, the link between MAC and cause-specific mortality has not yet been widely explored among obese and non-obese populations in the US. In the present study, we used data from NHANES III to investigate the relationship between MAC and the risk of all-cause, cardiovascular, and cancer mortality among obese and non-obese individuals in the US.

## Results

The study population comprised the 11,958 adults in the NHANES III database whose MAC measurements were available. Table 1 summarizes the clinical characteristics of each MAC tertile within the normal weight population. Participants in higher MAC tertile tended to have higher BMI, higher systolic and diastolic blood pressure, higher serum triglyceride levels, lower serum HDL levels, higher serum glucose levels, higher serum uric acid levels, higher serum AST levels, and higher serum total bilirubin levels than those in lower MAC tertile. They were also less likely to be non-Hispanic white, and more likely to be male, and to smoke than those in the lower MAC tertile. Table 2 summarizes the clinical characteristics of each MAC tertile within the overweight population. Participants in higher MAC tertile tended to have higher BMI, higher systolic and diastolic blood pressure, higher serum triglyceride levels, lower serum HDL levels, lower serum C-reactive protein levels, higher serum uric acid levels, higher serum AST levels, and higher serum total bilirubin levels than those in lower MAC tertile. They were also less likely to be non-Hispanic white, and more likely to be male and to smoke than those in the lower MAC tertile. The clinical characteristics of each MAC tertile within the obese population are summarized in Table 3. Participants with higher MAC tertile tended to have higher BMI, higher systolic and diastolic blood pressure, lower serum HDL levels, higher serum C-reactive protein levels, and higher serum uric acid levels than those in lower MAC tertile. They were also more likely to have type 2 diabetes mellitus.

**Table 1:** Characteristics of normal weight (19 ≤ BMI < 25) study participants.

Characteristic	Tertiles of mid-arm circumference (cm)			Total *n* = 4560	p-value
	T1 (18.0–27.2 cm) *n* = 1553	T2 (27.3–29.5 cm) *n* = 1515	T3 (29.6–42.0 cm) *n* = 1492		
Continuous variables					
Mid-arm circumference (cm), mean (SD)	25.68 (1.29)	28.49 (0.68)	31.31 (1.36)	28.46 (2.57)	<0.001
Age (years), mean (SD)	45.22 (21.95)	45.36 (19.89)	42.16 (16.97)	44.26 (19.79)	<0.001
BMI (kg/m^2^), mean (SD)	20.99 (1.52)	22.60 (1.39)	23.50 (1.16)	22.35 (1.72)	<0.001
Systolic blood pressure (mmHg), mean (SD)	118.64 (24.03)	121.66 (21.68)	122.29 (18.44)	120.85 (21.59)	<0.001
Diastolic blood pressure (mmHg), mean (SD)	66.58 (13.81)	69.03 (13.05)	72.47 (12.69)	69.33 (13.41)	<0.001
Serum triglycerides (mg/dL), mean (SD)	103.63 (76.31)	111.94 (77.67)	122.93 (92.16)	112.71 (82.63)	<0.001
HDL (mg/dL), mean (SD)	59.05 (16.00)	56.29 (16.75)	52.35 (15.14)	55.94 (16.21)	<0.001
Serum glucose (mg/dL), mean (SD)	91.73 (27.43)	93.35 (27.06)	95.21 (27.84)	93.41 (27.48)	0.002
C-reactive protein (mg/dL), mean (SD)	0.39 (0.69)	0.39 (0.88)	0.35 (0.62)	0.38 (0.74)	0.243
Serum uric acid (mg/dL), mean (SD)	4.46 (1.29)	4.85 (1.30)	5.44 (1.25)	4.91 (1.34)	<0.001
AST (U/L), mean (SD)	20.72 (12.80)	21.55 (18.61)	22.74 (13.02)	21.66 (15.06)	<0.001
Serum total bilirubin (mg/dL), mean (SD)	0.57 (0.37)	0.61 (0.35)	0.66 (0.33)	0.61 (0.35)	<0.001
Categorical variables					
Male, n (%)	317 (20.4)	656 (43.3)	1195 (80.1)	2168 (47.5)	<0.001
Non-Hispanic white, n (%)	722 (46.5)	704 (46.5)	630 (42.2)	2056 (45.1)	<0.001
Type 2 diabetes mellitus, n (%)	56 (3.6)	57 (3.8)	54 (3.6)	167 (3.7)	0.736
Malignancy, n (%)	65 (4.2)	46 (3.0)	42 (2.8)	153 (3.4)	0.008
Stroke, n (%)	30 (1.9)	28 (1.8)	31 (2.1)	89 (2.0)	0.876
Congestive heart failure, n (%)	37 (2.4)	28 (1.8)	24 (1.6)	89 (2.0)	0.555
Smoking, n (%)	72 (4.6)	119 (7.9)	230 (15.4)	421 (9.2)	<0.001
Physical activity, n (%)					<0.001
Ideal	470 (30.3)	530 (35.0)	532 (35.7)	1532 (33.6)	
Intermediate	709 (45.7)	739 (48.8)	761 (51.0)	2209 (48.4)	
None	374 (24.1)	246 (16.2)	199 (13.3)	819 (18.0)	

AST, aspartate aminotransferases; BMI, body mass index; HDL, High-density lipoprotein; SD, standard deviation.

**Table 2:** Characteristics of overweight (25 ≤ BMI < 30) study participants.

Characteristic	Tertiles of mid-arm circumference (cm)			Total *n* = 4438	p-value
	T1 (20.0–31.3 cm) *n* = 1501	T2 (31.4–33.4 cm) *n* = 1499	T3 (33.5–52.0 cm) *n* = 1438		
Continuous variables					
Mid-arm circumference (cm), mean (SD)	29.93 (1.26)	32.52 (0.62)	35.14 (1.34)	32.49 (2.39)	<0.001
Age (years), mean (SD)	52.84 (20.92)	49.42 (18.20)	45.44 (16.25)	49.29 (18.82)	<0.001
BMI (kg/m^2^), mean (SD)	26.57 (1.21)	27.28 (1.40)	28.00 (1.35)	27.28 (1.44)	<0.001
Systolic blood pressure (mmHg), mean (SD)	128.09 (23.78)	126.90 (21.27)	125.84 (19.09)	126.96 (21.51)	0.020
Diastolic blood pressure (mmHg), mean (SD)	71.80 (12.15)	73.99 (12.35)	75.47 (11.73)	73.73 (12.18)	<0.001
Serum triglycerides (mg/dL), mean (SD)	154.07 (113.78)	154.72 (108.12)	160.09 (126.93)	156.24 (116.41)	0.031
HDL (mg/dL), mean (SD)	50.95 (14.42)	48.35 (14.27)	46.34 (12.62)	48.58 (13.94)	<0.001
Serum glucose (mg/dL), mean (SD)	100.65 (37.29)	101.10 (36.28)	101.92 (36.31)	101.22 (36.63)	0.637
C-reactive protein (mg/dL), mean (SD)	0.48 (0.77)	0.44 (0.66)	0.38 (0.75)	0.43 (0.73)	0.001
Serum uric acid (mg/dL), mean (SD)	5.17 (1.40)	5.57 (1.45)	5.90 (1.36)	5.54 (1.44)	<0.001
AST (U/L), mean (SD)	21.47 (14.56)	22.31 (13.73)	23.84 (14.85)	22.52 (14.41)	<0.001
Serum total bilirubin (mg/dL), mean (SD)	0.57 (0.32)	0.61 (0.32)	0.64 (0.36)	0.61 (0.34)	<0.001
Categorical variables					
Male, n (%)	502 (33.4)	877 (58.5)	1089 (75.7)	2468 (55.6)	<0.001
Non-Hispanic white, n (%)	627 (41.8)	648 (43.2)	557 (38.7)	1832 (41.3)	<0.001
Type 2 diabetes mellitus, n (%)	130 (8.7)	123 (8.2)	111 (7.7)	364 (8.2)	0.453
Malignancy, n (%)	63 (4.2)	49 (3.3)	31 (2.2)	143 (3.2)	0.008
Stroke, n (%)	48 (3.2)	45 (3.0)	25 (1.7)	118 (2.7)	0.187
Congestive heart failure, n (%)	50 (3.3)	57 (3.8)	33 (2.3)	140 (3.2)	0.156
Smoking, n (%)	113 (7.5)	216 (14.4)	260 (18.1)	589 (13.3)	<0.001
Physical activity, n (%)					<0.001
Ideal	437 (29.1)	485 (32.4)	484 (33.7)	1406 (31.7)	
Intermediate	695 (46.3)	739 (49.3)	767 (53.3)	2201 (49.6)	
None	369 (24.6)	275 (18.3)	187 (13.0)	831 (18.7)	

AST, aspartate aminotransferases; BMI, body mass index; HDL, High-density lipoprotein; SD, standard deviation.

**Table 3:** Characteristics of obese (BMI ≥ 30) study participants.

Characteristic	Tertiles of mid-arm circumference (cm)			Total *n* = 2960	p-value
	T1 (28.0–35.4 cm) *n* = 1009	T2 (35.5–38.2 cm) *n* = 947	T3 (38.3–61.0 cm) *n* = 1004		
Continuous variables					
Mid-arm circumference (cm), mean (SD)	33.70 (1.45)	36.88 (0.78)	41.48 (3.01)	37.36 (3.79)	<0.001
Age (years), mean (SD)	50.26 (17.88)	47.33 (15.84)	45.00 (15.44)	47.54 (16.57)	<0.001
BMI (kg/m^2^), mean (SD)	32.18 (1.99)	33.44 (2.63)	38.42 (5.33)	34.70 (4.53)	<0.001
Systolic blood pressure (mmHg), mean (SD)	128.20 (22.11)	128.42 (19.93)	130.84 (19.85)	129.17 (20.70)	0.008
Diastolic blood pressure (mmHg), mean (SD)	74.40 (12.61)	76.37 (12.19)	77.93 (12.89)	76.23 (12.65)	<0.001
Serum triglycerides (mg/dL), mean (SD)	173.76 (130.33)	184.20 (142.86)	170.46 (121.57)	175.99 (131.75)	0.057
HDL (mg/dL), mean (SD)	48.33 (13.58)	46.25 (14.25)	44.97 (12.63)	46.53 (13.56)	<0.001
Serum glucose (mg/dL), mean (SD)	107.02 (45.42)	109.11 (45.62)	110.71 (47.88)	108.94 (46.34)	0.201
C-reactive protein (mg/dL), mean (SD)	0.58 (0.75)	0.56 (0.71)	0.75 (0.84)	0.63 (0.77)	<0.001
Serum uric acid (mg/dL), mean (SD)	5.48 (1.53)	5.85 (1.49)	6.03 (1.54)	5.79 (1.54)	<0.001
AST (U/L), mean (SD)	23.41 (17.81)	23.53 (14.27)	23.41(13.83)	23.45(15.43)	0.982
Serum total bilirubin (mg/dL), mean (SD)	0.54 (0.28)	0.56 (0.28)	0.53 (0.29)	0.54 (0.28)	0.204
Categorical variables					
Male, n (%)	290 (28.7)	458 (48.4)	398 (39.6)	1146 (38.7)	<0.001
Non-Hispanic white, n (%)	353 (35.0)	346 (36.5)	321 (32.0)	1020 (34.5)	<0.001
Type 2 diabetes mellitus, n (%)	100 (9.9)	108 (11.4)	136 (13.5)	344 (11.6)	0.007
Malignancy, n (%)	44 (4.4)	28 (3.0)	25 (2.5)	97 (3.3)	0.050
Stroke, n (%)	21 (2.1)	21 (2.2)	24 (2.4)	66 (2.2)	0.895
Congestive heart failure, n (%)	32 (3.2)	22 (2.3)	38 (3.8)	92 (3.1)	0.460
Smoking, n (%)	68 (6.7)	122 (12.9)	99 (9.9)	289 (9.8)	<0.001
Physical activity, n (%)					<0.001
Ideal	245 (24.3)	238 (25.1)	221 (22.0)	704 (23.8)	
Intermediate	476 (47.2)	511 (54.0)	546 (54.4)	1533 (51.8)	
None	288 (28.5)	198 (20.9)	237 (23.6)	727 (24.4)	

AST, aspartate aminotransferases; BMI, body mass index; HDL, High-density lipoprotein; SD, standard deviation.

In the present study, the median length of follow-up was 14.3 years. With regard to anthropometric parameters associated with all-cause mortality (shown in Table 4), the unadjusted hazard ratio (HR) for increasing MAC in Model 1 was 0.97 (95% confidence interval [CI]: 0.96–0.98). The multivariate-adjusted HRs in Models 2, 3, and 4 for increasing MAC were 0.95 (95% CI: 0.95–0.97), 0.95 (95% CI: 0.93–0.97), and 0.94 (95% CI: 0.92–0.96), respectively. This result indicates that MAC is inversely associated with all-cause mortality among the general population in the US. An inverse association between MAC and the risk of all-cause mortality was observed in non-obese subjects, with an up to 28% lower HR for all-cause mortality in normal weight participants and 24% lower HR for all-cause mortality in overweight participants, but not in obese subjects (Table 5). There were no apparent relationships between MAC and cardiovascular (Table 6) or cancer mortality (Table 7) in either the non-obese or the obese subjects after multiple-covariate adjustment.

**Table 4:** Cox proportional-hazards regression of all-cause mortality for different anthropometric parameters and BMI in US individuals.

Anthropometric Parameters	Model^a^ 1 HR (95% CI)	Model^a^ 2 HR (95% CI)	Model^a^ 3 HR (95% CI)	Model^a^ 4 HR (95% CI)
MAC	0.97 (0.96, 0.98)	0.95 (0.93, 0.97)	0.94 (0.93, 0.97)	0.94 (0.92, 0.96)
WC^a^	1.02 (1.01, 1.02)	1.01 (1.00, 1.02)	1.00 (0.99, 1.01)	1.00 (0.99, 1.01)
WHR	2.93 (1.89, 6.78)	2.91 (1.59, 5.34)	1.96 (1.06, 3.63)	1.57 (0.85, 2.91)
TC	0.92 (0.91, 0.93)	0.96 (0.95, 0.98)	0.97 (0.96, 0.98)	0.98 (0.96, 0.99)
BMI	1.00 (0.99,1.00)	1.00 (0.99,1.01)	0.98 (0.97,0.99)	0.98 (0.97, 0.99)

^a^Adjusted covariates Model 1: Unadjusted Model 2: Adjustment for age, race, sex and BMI Model 3: Model 2 + serum triglycerides, serum HDL, serum glucose, serum aspartate transaminase, C-reactive protein, serum uric acid, and serum total bilirubin Model 4: Model 3 + physical activity, systolic blood pressure, smoking, and type 2 diabetes mellitus MAC: mid-arm circumference WC: waist circumference WHR: waist hip ratio TC: thigh circumference BMI: body mass index.

**Table 5:** Cox proportional-hazards regression of all-cause mortality for mid-arm circumference in normal weight, overweight and obese US individuals.

Models^a^	Tertiles of MAC	Normal weight	Overweight	Obesity
		Hazard Ratio (95% CI)	Hazard Ratio (95%CI)	Hazard Ratio (95%CI)
**Model 1**	T2 *vs*. T1	0.83 (0.72–0.97)	0.67 (0.58–0.77)	0.86 (0.70–1.05)
	T3 *vs*. T1	0.62 (0.52–0.72)	0.48 (0.41–0.56)	0.87 (0.71–1.05)
**Model 2**	T2 *vs*. T1	0.73 (0.61–0.87)	0.83 (0.72–0.97)	1.02 (0.83–1.25)
	T3 *vs*. T1	0.68 (0.55–0.85)	0.82 (0.69–0.99)	1.18 (0.92–1.52)
**Model 3**	T2 *vs*. T1	0.74 (0.62–0.89)	0.84 (0.72–0.97)	0.97 (0.79–1.12)
	T3 *vs*. T1	0.70 (0.56–0.87)	0.79 (0.65–0.95)	1.12 (0.87–1.45)
**Model 4**	T2 *vs*. T1	0.76 (0.64–0.91)	0.81 (0.70–0.95)	0.97 (0.79–1.19)
	T3 *vs*. T1	0.72 (0.58–0.90)	0.76 (0.63–0.91)	1.11 (0.86–1.43)

^a^Adjusted covariates: Model 1: Unadjusted Model 2: Adjustment for age, race, sex and BMI Model 3: Model 2 + serum triglycerides, serum HDL, serum glucose, serum aspartate transaminase, C-reactive protein, serum uric acid, and serum total bilirubin Model 4: Model 3 + physical activity, systolic blood pressure, smoking, and type 2 diabetes mellitus.

**Table 6:** Cox proportional-hazards regression of cardiovascular mortality for mid-arm circumference in normal weight, overweight and obese US individuals.

Models^a^	Tertiles of MAC	Normal weight	Overweight	Obesity
		Hazard Ratio (95% CI)	Hazard Ratio (95%CI)	Hazard Ratio (95%CI)
**Model 1**	T2 *vs*. T1	0.54 (0.30–0.96)	0.59 (0.33–1.04)	0.90 (0.68–1.20)
	T3 *vs*. T1	0.46 (0.26–0.80)	0.52 (0.34–1.02)	0.81 (0.60–1.08)
**Model 2**	T2 *vs*. T1	1.06 (0.58–1.93)	1.01 (0.59–1.93)	1.18 (0.87–1.58)
	T3 *vs*. T1	0.69 (0.36–1.30)	0.73 (0.40–1.35)	1.34 (0.92–1.95)
**Model 3**	T2 *vs*. T1	0.94 (0.51–1.72)	0.96 (0.53–1.74)	1.11 (0.82–1.50)
	T3 *vs*. T1	0.62 (0.33–1.16)	0.66 (0.36–1.22)	1.28 (0.87–1.86)
**Model 4**	T2 *vs*. T1	0.94 (0.52–1.71)	0.93 (0.52–1.70)	1.13 (0.83–1.53)
	T3 *vs*. T1	0.60 (0.39–1.13)	0.66 (0.35–1.18)	1.19 (0.81–1.75)

^a^Adjusted covariates: Model 1: Unadjusted. Model 2: Adjustment for age, race, sex and BMI. Model 3: Model 2 + serum triglycerides, serum HDL, serum glucose, serum aspartate transaminase, C-reactive protein, serum uric acid, and serum total bilirubin. Model 4: Model 3 + physical activity, systolic blood pressure, smoking, type 2 diabetes mellitus.

**Table 7:** Cox proportional-hazards regression of cancer mortality for mid-arm circumference in normal weight, overweight and obese US individuals.

Models^a^	Tertiles of MAC	Normal weight	Overweight	Obesity
		Hazard Ratio (95% CI)	Hazard Ratio (95%CI)	Hazard Ratio (95%CI)
**Model 1**	T2 *vs*. T1	1.22 (0.38–3.90)	0.29 (0.02–4.66)	0.61 (0.41–0.92)
	T3 *vs*. T1	1.07 (0.34–3.37)	0.22 (0.03–1.61)	0.77 (0.53–1.13)
**Model 2**	T2 *vs*. T1	2.07 (0.63–6.76)	0.97 (0.06–17.28)	0.76 (0.50–1.17)
	T3 *vs*. T1	1.59 (0.47–5.38)	2.19 (0.92–17.83)	1.17 (0.71–1.95)
**Model 3**	T2 *vs*. T1	1.77 (0.56–4.33)	1.18 (0.65–21.49)	0.75 (0.49–1.14)
	T3 *vs*. T1	1.36 (0.41–3.12)	2.70 (0.87–23.04)	1.12 (0.67–1.86)
**Model 4**	T2 *vs*. T1	1.77 (0.54–5.89)	1.17 (0.06–22.67)	0.75 (0.49–1.15)
	T3 *vs*. T1	1.38 (0.41–4.71)	2.42 (0.27–21.60)	1.08 (0.64–1.80)

^a^Adjusted covariates: Model 1: Unadjusted Model 2: Adjustment for age, race, sex and BMI Model 3: Model 2 + serum triglycerides, serum HDL, serum glucose, serum aspartate transaminase, C-reactive protein, serum uric acid, and serum total bilirubin Model 4: Model 3 + physical activity, systolic blood pressure, smoking, and type 2 diabetes mellitus.

## Discussion

In the present study, conducted in the general population in the US, we determined whether there is an association between MAC and all-cause, cardiovascular, and cancer mortality risk; the link between MAC and cause-specific mortality in US obese and non-obese participants had not previously been comprehensively evaluated. The results showed an inverse association between MAC and all-cause mortality in non-obese participants, after various multiple-covariate adjustment strategies, with an up to 28% lower HR for all-cause mortality. There was no such association in obese participants. These findings may have substantial additional clinical implications for death risk prediction, whereby the benefits of MAC may be compromised by the detrimental effects of obesity on all-cause mortality.

MAC has been seen as a valuable anthropometric marker of assessing undernutrition and obesity in children and adolescents^2–4^. Several previous studies have highlighted the predictive value of MAC on all-cause, and cause-specific mortality in both Western and Asian participants^12–17^. Using data from the British National Diet and Nutrition Survey, which followed up 1,054 individuals aged ≥65 years for more than 15 years, Bates *et al*. demonstrated that each standard deviation (3.3 cm) rise in MAC was associated with a reduced overall mortality risk of 15%^12^. In addition, in another study involving elderly subjects, Wijnhoven and his colleagues^13^ reported that the HR for mortality in each lower SD of MAC was 2.26 (95% CI: 1.71–3.00) in women and 1.79 (1.48–2.16) in men. In addition, Chen *et al*. reported that MAC was a predictor of cause-specific mortality in a cohort study involving 19,575 adults in Bangladesh^14^. They reported that MAC was inversely correlated with the risk of all-cause and cardiovascular death, and with cardiovascular disease, but not with cancer mortality. These studies indicate that MAC plays a critical role in all-cause and cardiovascular mortality; our own results partially corroborate these previous reports by revealing that MAC may be a predictor of all-cause mortality in non-obese (BMI level: <30) participants in the US.

There are several mechanisms that may mediate the inverse relationship between MAC and all-cause mortality risk in non-obese individuals; for instance, deterioration of fat-free or total muscle mass, impairment of lipid and glucose metabolism, diminished nutritional status, or malnutrition. Firstly, deterioration of fat-free or total muscle mass, which may contribute to reduced MAC, has been linked to higher mortality, as well as to a number of physiological factors and underlying medical illnesses^5, 6, 18^. For instance, Visser *et al*.^18^ reported that higher fat infiltration within muscle tissue, as well as lower muscle mass, was linked to impaired mobility in elderly subjects. Poor muscle strength was associated with disability in activities of daily living^19^, and reduced physical activity predicted a higher risk of losing muscle mass and strength^20, 21^. Several underlying chronic conditions (*e.g*., type 2 diabetes mellitus, stroke, coronary heart disease, and chronic obstructive pulmonary disease) have also been associated with lower muscle mass and strength^22^. Illness-related muscle deterioration from physical inactivity, systemic inflammation, and nutritional depletion may be associated with higher mortality rates^23–26^. Secondly, impairment of lipid and glucose metabolism may play a significant role in the inverse association between MAC and the risk of all-cause mortality. In this regard, Hegarty *et al*. thoroughly examined the correlation between insulin resistance and intramuscular lipids^27^, and Olsen *et al*. demonstrated that arm muscles have better insulin sensitivity and higher insulin-mediated glucose clearance than leg muscles^28^. Thirdly, diminished nutrition or severe malnourishment, which is directly proportional to MAC, can be linked to an increase in the risk of mortality among infants and children^29–31^. Therefore, it is important to detect malnutrition early, and MAC may be an efficacious and non-invasive tool to assess nutritional status and identify subjects with a high risk of death. Such studies indicate why MAC plays such an important role in predicting all-cause mortality in non-obese individuals.

However, in the present study, the benefits of MAC-which can be used to estimate peripheral subcutaneous adipose tissue load-were compromised by the detrimental effects of obesity on all-cause mortality. There are several possible explanations for such an effect. For instance, the abdominal or visceral fat in obese individuals may cause higher rates of lipolysis, leading in turn to the release of free fatty acids into the circulation, as well as to insulin resistance, atherosclerosis, and type 2 diabetes mellitus, all of which are correlated with an elevated risk of mortality^32, 33^. Relatedly, recent studies^34–36^ have shown that body fat distribution is relevant to mortality risk, because subcutaneous adipose tissue releases difference levels of anti-inflammatory adipokines, circulating adiponectin, and cytokines, which may play a role in the survival benefits to non-obese individuals.

The present study, which extends the traditional application of MAC for identifying obesity or undernutrition in children and adolescents, provides evidence for an inverse association between MAC and all-cause mortality in non-obese individuals in the US. These findings emphasize the importance of MAC measurement as a substantial additional and clinical predictor of non-obese individuals who are at risk of all-cause mortality.

This study had several limitations. Firstly, the MAC was measured only once during the follow-up period, which may have led to biased results; likewise, the use of self-reported variables may have contributed to reporting bias. Secondly, the NHANES III was an observational survey that was conducted at a single time point, rather than an analysis of long-term repeated observations. Therefore, we were unable to evaluate causality in the relationship between MAC and the risk of cause-specific mortality, which would necessitate repeated MAC measurements over time. Thirdly, we cannot rule out the possibility that some confounding factors in the relationship between MAC and the risk of cause-specific mortality in obese and non-obese individuals remained unmeasured, even though we adjusted for a large number of potentially confounding variables. Several confounding factors, including different type and level of physical activity, may change the risks for mortality. We observed little overlap of MAC level between the non-obese and obese tertiles. The difference level of physical activity between these two groups could have influenced the main results.

In conclusion, we identified that MAC had good predictive ability in predicting follow-up mortality risk of non-obese population in the US. Findings from prior studies demonstrate that MAC may be an effective tool for anthropometric measure and nutritional assessment; however, our study indicated that, in non-obese participants, a significant inverse association exists between MAC and risk of all-cause mortality and higher MAC might provide a beneficial effect. These findings strengthened the importance of using anthropometric assessment tools and indicated that additional studies were warranted to clarify the mechanisms that mediate this relationship.

## Materials and Methods

### Study Population

Our study sample was representative of the US population and included participants aged 20–90 years, who underwent a survey and a health examination. This constituted a complex sample representing the US non-institutionalized population from NHANES III, 1988 to 1994; details of the survey design and collection procedures have been reported previously^37^. Demographic data collection in the NHANES III consisted of several components: a structured home interview, physical examination, blood sampling, anthropometric measurement, and body-composition assessment. All participants in the NHANES III provided written informed consent, and the study was approved by National Center for Health Statistics (NCHS) Institutional Review Board before it began; it was executed in accordance with the Declaration of Helsinki.

### Mid-Arm Circumference Tertiles-based Subgroups

The participants’ MACs were measured using the standard procedures described by Lohman *et al*., which depend on the age of the participant^38^. The participants were divided into three groups on the basis of BMI: 19 ≤ BMI < 25 kg/m^2^ (normal weight group), 25 ≤ BMI < 30 kg/m^2^ (overweight group) and BMI ≥30 kg/m^2^ (obesity group); each group was then divided into three subgroups depending on their MAC level. The tertiles were as follows: T1 (18.0–27.2), T2 (27.3–29.5), T3 (29.6–42.0) cm in the normal weight group, T1 (20.0–31.3), T2 (31.4–33.4), T3 (33.5–52.0) cm in the overweight group and T1 (28.0–35.4), T2 (35.5–38.2), T3 (38.3–61.0) cm in the obesity group.

### Follow-up Data on All-Cause, Cardiovascular, and Cancer Mortality

Follow-up data were collected regarding the all-cause, cardiovascular, and cancer mortality of the NHANES III study participants from 1988 to 2006; these data included detailed mortality information from the time of study participation^39^. The data were provided by the NCHS on the basis of probabilistic matching between the NHANES III participants and the National Death Index death certificate records.

### Covariates

The following details were self-reported collected in the NHANES III: age, gender, race/ethnicity, medical conditions (type 2 diabetes mellitus, malignancy, stroke, and congestive heart failure), smoking status, and physical activity. Type 2 diabetes mellitus was self-reported on the basis of a physician’s diagnosis, diagnosed on the basis of random serum glucose level (≥200 mg/dL), or inferred from the participant’s use of diabetic medications (insulin injections and/or oral hypoglycemic agents). An NHANES-associated physician measured systolic and diastolic blood pressure three or four times using a mercury sphygmomanometer, and the average of these readings was used. The serum biochemical profiles were measured at the Lipoprotein Analytical Laboratory at Johns Hopkins University, Baltimore, Maryland. Specifically, serum uric acid level was measured using a Hitachi 737 automated multichannel chemistry analyzer (Boehringer Mannheim Diagnostics, Indianapolis, IN, USA). All measurements were conducted using standardized methods whose accuracy had been documented by the Centre for Disease Control and Prevention (CDC)^37, 38^.

### Physical activity

Participants were asked the following leisure time physical activities, which and how frequently they engaged in during the past month: riding a bicycle, swimming, aerobic or other dance, jogging or running (≥1 mile), callisthenic or floor exercise, weight lifting and gardening or yard work. Duration of the physical activity was not ascertained in NHANES III.

Based on the physical activity intensity value (metabolic equivalent tasks [METs]), which represents the ratio of the energy expenditure of the activity to the basal metabolic rate, the participants classified as ideal, intermediate, and none, respectively^40^. For instance, the participants was classified as physically active (ideal category) if they engaged in any physical activity with 6 or more METs and 3.0 or more times per week, or any physical activity with 3 to 5.9 METs and 5 or more times per week, meaning that 150 minutes per week or more at moderate vigorous intensity, or 150 minutes per week or more at moderate + vigorous intensity, or 75 minutes per week or more at vigorous intensity. Participants were classified as intermediate category in physical activity: 1 to 149 minutes per week at moderate intensity, or 1 to 149 minutes per week at moderate + vigorous intensity, or 1 to 74 minutes per week at vigorous intensity.

### Data Analysis

All statistics were analyzed using SPSS version 18 (SPSS Inc., Chicago, IL); the analyses incorporated sampling weights, and incorrect estimates of variance were prevented using the Complex Samples procedure. We conducted a complete case analysis in the present study. Quantitative variables were summarized in terms of their means and standard deviations (SD), while qualitative variables were summarized in terms of their frequency and percentages. Differences in demographic characteristics were tested using the independent t-test or Wilcoxon Rank sum test (for continuous variables), or the Chi-square test (for discrete variables). Two-sided p-values of less than 0.05 were regarded as indicating statistical significance. Multivariate Cox proportional hazard models were used to evaluate the association between the MAC tertiles in obese or non-obese subjects, and all-cause, cardiovascular, and cancer mortality. An extended-model approach was used adjust for covariates: Model 1 was not adjusted for any other variables; Model 2 was further adjusted for age, race, sex, and BMI; Model 3 was adjusted for all the additional variables that were in Model 2, as well as for serum triglycerides, serum HDL, serum glucose, serum aspartate transaminase, C-reactive protein, serum uric acid, and serum total bilirubin. Model 4 was similar to Model 3, but the following additional factors were also adjusted for: physical activity, systolic blood pressure, smoking, and type 2 diabetes mellitus.
